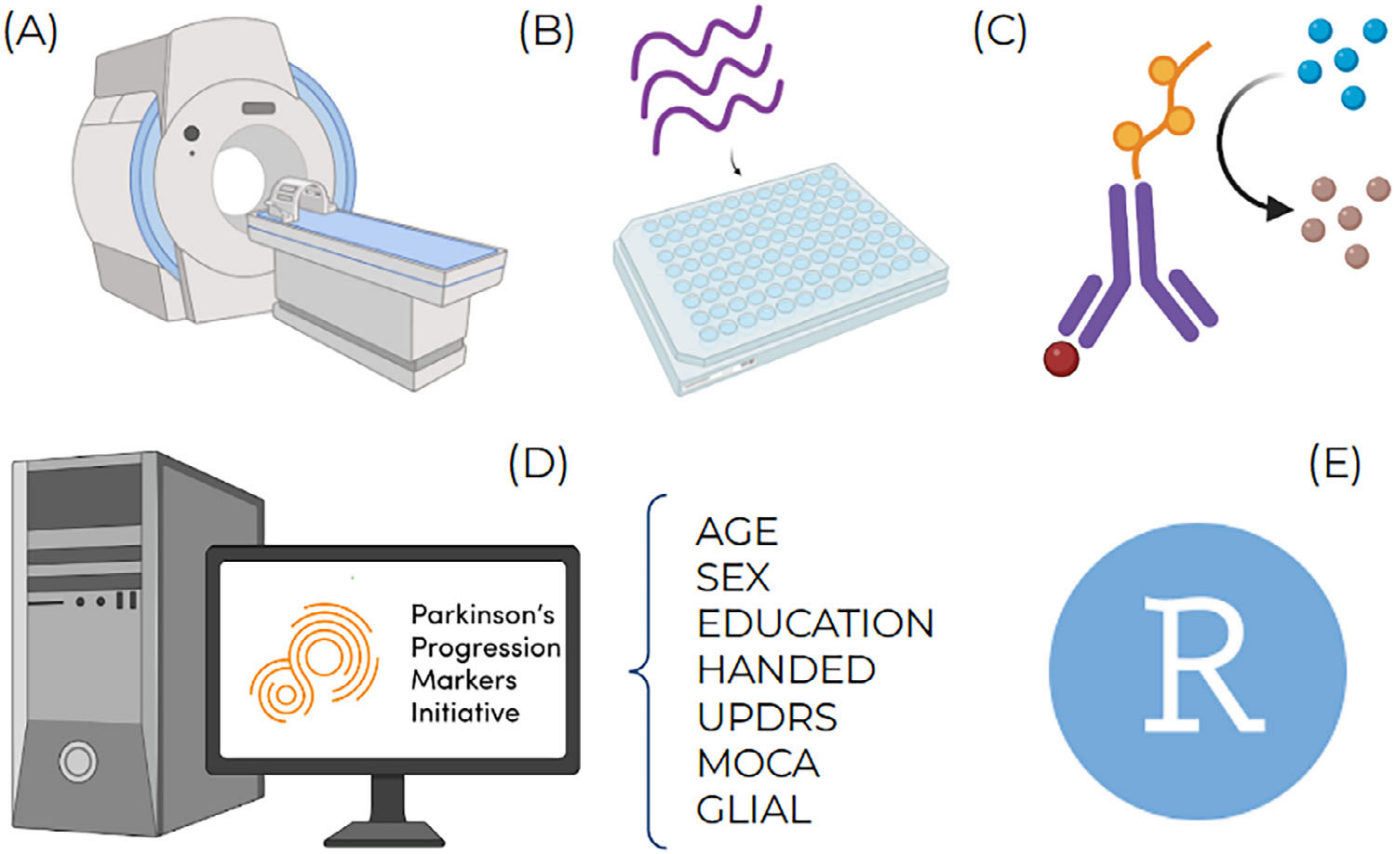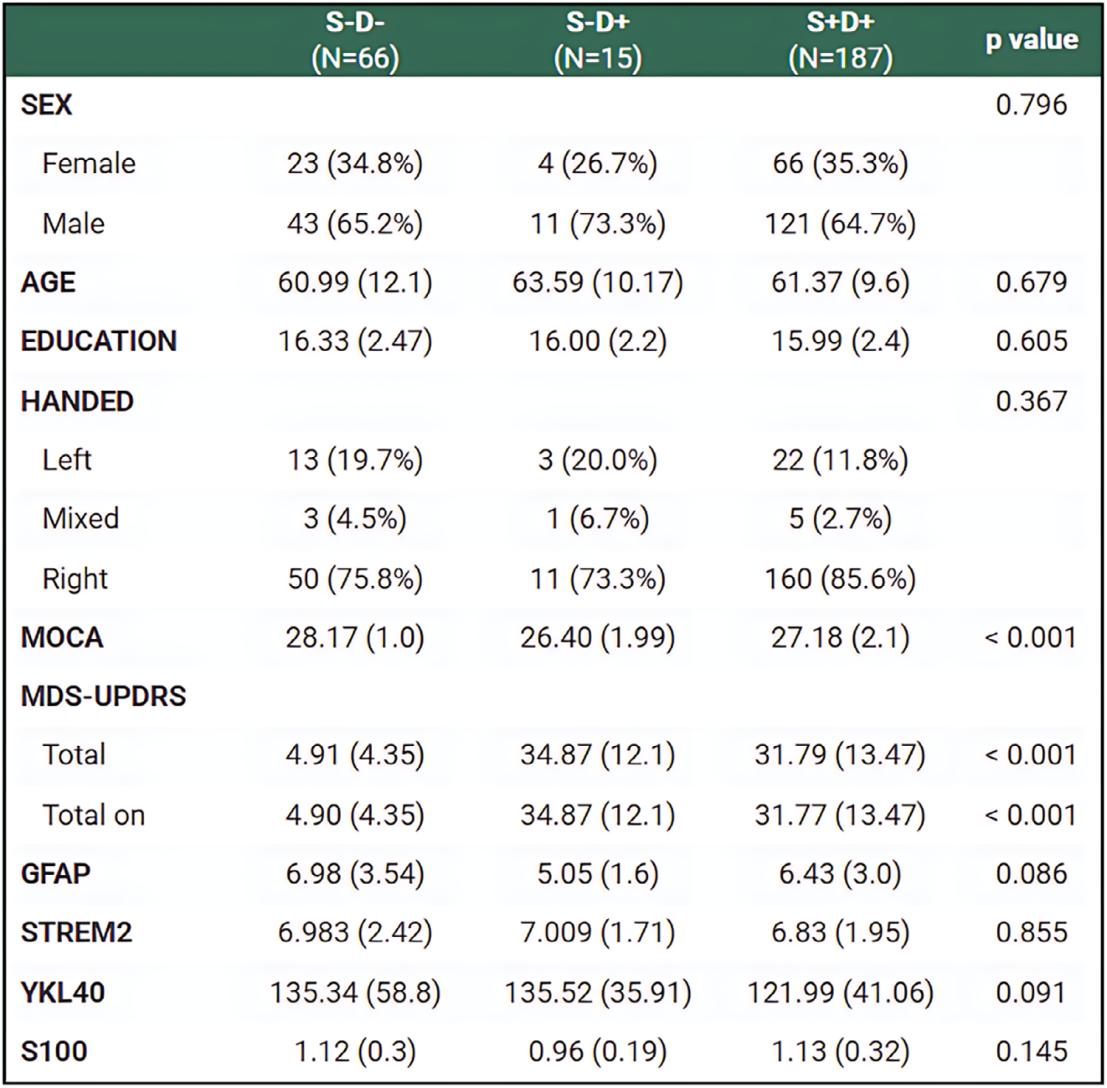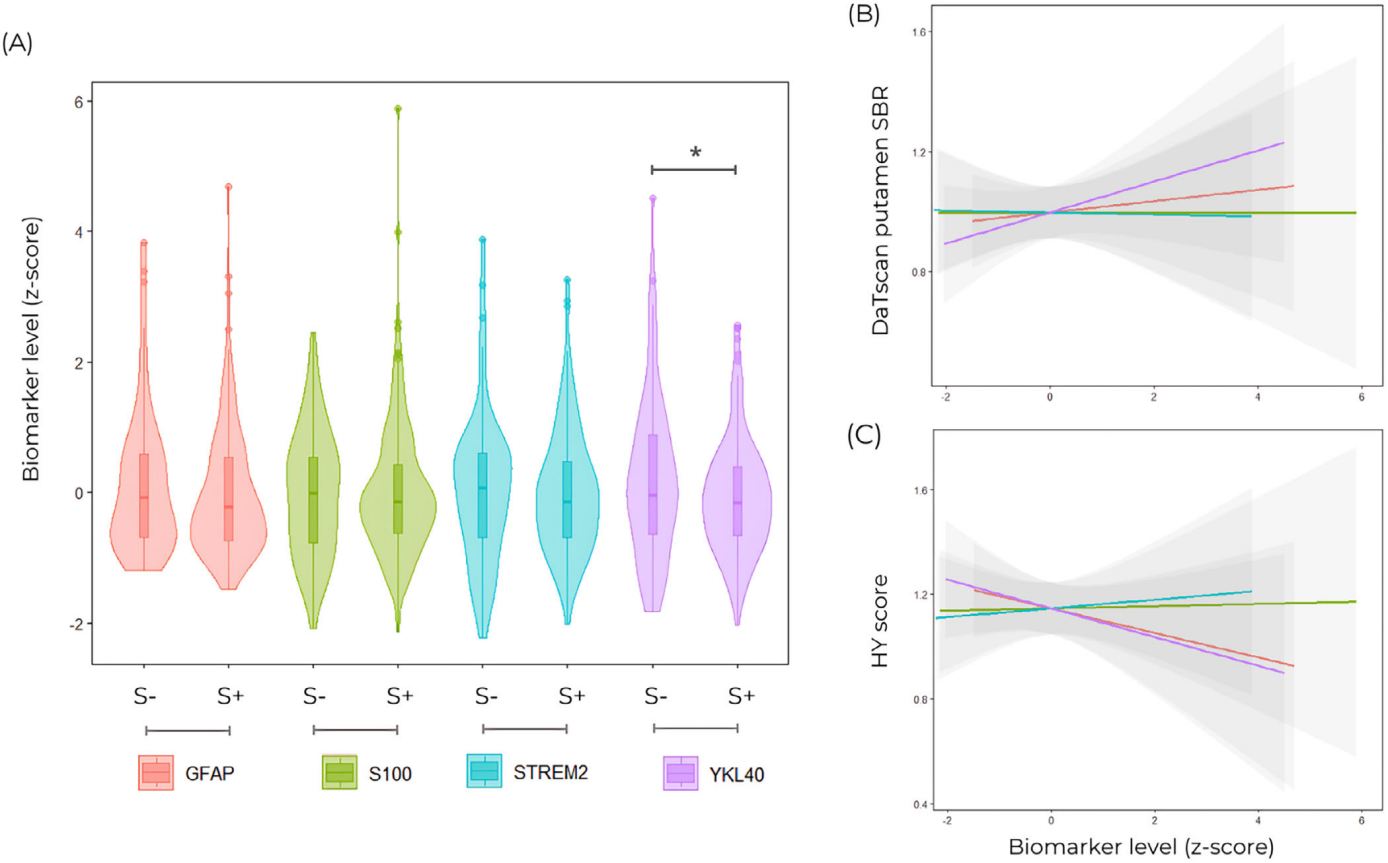# Glial interaction with Parkinsonian disorders biomarkers: alpha‐synuclein, dopaminergic dysfunction and clinical stages

**DOI:** 10.1002/alz70856_106159

**Published:** 2026-01-08

**Authors:** Lara Angi Souza, Daniel Teixeira dos Santos, Eduardo R. Zimmer, Wyllians Vendramini Borelli, Artur Francisco Schumacher‐Schuh

**Affiliations:** ^1^ Federal University of Rio Grande do Sul, Porto Alegre, Rio Grande do Sul, Brazil, Porto Alegre, RS, Brazil; ^2^ Neurology Department, Hospital de Clínicas de Porto Alegre, Porto Alegre, Rio Grande do Sul, Brazil; ^3^ Neurology Department, Santa Casa de Misericórdia de Porto Alegre, Porto Alegre, Rio Grande do Sul, Brazil; ^4^ Federal University of Rio Grande do Sul (UFRGS), Porto Alegre, RS, Brazil; ^5^ Brain Institute of Rio Grande do Sul (InsCer), PUCRS, Porto Alegre, Rio Grande do Sul, Brazil; ^6^ McGill Centre for Studies in Aging, Montreal, QC, Canada; ^7^ Universidade Federal do Rio Grande do Sul, Porto Alegre, Rio Grande do Sul, Brazil; ^8^ Centro de Memória, Hospital Moinhos de Vento, Porto Alegre, RS, Brazil; ^9^ Clinical Hospital of Porto Alegre, Porto Alegre, Rio Grande do Sul, Brazil; ^10^ Hospital de Clínicas de Porto Alegre, Porto Alegre, RS, Brazil

## Abstract

**Background:**

The neuronal α‐Synuclein disease (NSD) has been recently proposed to identify biological anchors of Parkinson's disease, comprehending neuronal alpha‐synuclein (S) and dopaminergic neuronal dysfunction (D). However, interactions with glial cells are still poorly understood in the context of α‐syn pathology. In this study, we investigated the relationship between glial markers and PD pathophysiology, specifically to identify which glial markers have stronger association.

**Method:**

The Parkinson's Progression Markers Initiative (PPMI) database was used to retrieve cerebrospinal fluid (CSF) glial biomarkers measures. Biological anchor S was assessed by Amprion‐αS‐SAA (Figure 1a‐d) and D as assessed with DaTscan visual interpretation and Putamen's lowest SBR value. CSF GFAP, STREM2, YKL40 and S100 were measured using Roche NeuroTool Kit. Clinical and cognitive variables collected included sex, years of education, Hoehn & Yahr scale (HY) and the total MDS‐UPDRS data. Group comparisons were analyzed using Chi‐squared test and ANOVA. General linear models adjusted for age were conducted to evaluate the association of glial markers, biological anchors, and HY. All statistical analyses were performed in R v4.

**Result:**

A total of 268 individuals (67 controls and 201 PD) were evaluated (mean age 61.4±10.3 years, Tab. 1). Compared with S‐D‐, S+D+ individuals had higher scoresworse levels of total MDS‐UPDRS (*p* < 0.001) and lower MOCA (*p* < 0.001) scores. CSF YKL‐40 levels were associated with S (beta = ‐0.37, *p* =  0.02) and D biological anchors (b = ‐0.37, *p* =  0.02, Figure 2b) adjusted for age. CSF GFAP, S100B and sTREM2 were not associated with S or D (*p* > 0.05). When evaluating clinical symptoms of PD with HY, CSF YKL‐40 was associated with HY scores (beta = ‐0.36, *p* =  0.02, Figure 2c). However, in S+ individuals, only CSF GFAP levels showed a significant interaction with DaT putamen SBP levels and HY scores (beta = 0.65, *p* =  0.004).

**Conclusion:**

CSF YKL‐40 may be an early event in PD's neuronal alpha‐synuclein pathological process. However, CSF GFAP levels may be associated with PD functional independence related to dopamine dysfunction in S+ individuals. Further studies may investigate the interaction between glial markers and biological anchors in PD.